# A fMRI neuroimaging dataset of word reading with semantic and phonological localizers in children and adolescents

**DOI:** 10.1016/j.dib.2025.112248

**Published:** 2025-11-08

**Authors:** Jiuru Wang, Avery Vess, Avantika Mathur, Neelima Wagley, David Quinto-Pozos, James R. Booth

**Affiliations:** a Department of Psychology and Human Development, Vanderbilt University, Nashville, TN, USA; b College of Health Solutions, Arizona State University, Tempe, AZ, USA; c Department of Linguistics, University of Texas at Austin, Austin, TX, USA

**Keywords:** fMRI, Neuroimaging, Reading, Semantic, Phonological, Children, Adolescents

## Abstract

Here we describe the public neuroimaging and behavioural dataset entitled “A fMRI neuroimaging dataset of word reading with semantic and phonological localizers in children and adolescents” available on the OpenNeuro project (https://openneuro.org). This dataset examines neural mechanisms of reading development in children and adolescents 10–17 years of age who have typical hearing (*N* = 83) as part of a larger longitudinal study involving deaf and hard-of-hearing (DHH) children. The data includes T1-weighted structural magnetic resonance imaging (sMRI), diffusion tensor imaging (DTI) and task based functional MRI (fMRI). In total, 83 children performed six fMRI tasks, including two reading tasks, one involving ‘visual word rhyming’ judgments and the other involving ‘visual word meaning’ judgments. The visual word rhyming task parametrically manipulated orthographic and phonological conflict and the visual word meaning task manipulated the strength of semantic association. Children also performed four ‘localizer’ tasks including ‘picture rhyming’, ‘picture meaning’, ‘speech reading’ involving matching lip movements, and ‘signed language’ involving matching handshape, location or movement. The use of localizers is innovative allowing one to independently identify regions involved in phonological and semantic processing without phonological or orthographic input to examine how these mechanisms may be involved in reading. There is also a comprehensive battery of standardized assessments that measures hearing, reading, language, and cognitive processes that enable detailed analyses of brain-behaviour relationships. Moreover, questionnaires were administered to comprehensively describe the developmental and health history of the participants.

Specifications Table

**Table:** 

Subject	Developmental and Educational Psychology Experimental and Cognitive Psychology [1]
Specific subject area	Neuroimaging for Reading and Language Development
Type of data	Tables, Images, Figures
Data collection	Data were collected using a 3 Tesla Philips Elition MRI scanner with advanced acquisition protocols, including structural, diffusion and functional MRI. Behavioural data were gathered using standardized reading, language, and cognitive assessments and questionnaires.
Data source location	Brain Development Lab, Vanderbilt University, Nashville, Tennessee, USA
Data accessibility	Repository name: OpenNeuro Data identification number: doi:10.18112/openneuro.ds006239.v1.0.2 Direct URL to data: https://openneuro.org/datasets/ds006239
Related research article	

## 1 Value of the Data

• Provides neuroimaging fMRI data from a large sample of children on two visual word reading tasks with parametric measures of orthographic/phonological and semantic processing
• Inclusion of four ‘localizer’ tasks enables independent identification of neural regions related to phonology, semantics, American Sign Language (ASL), and speech reading.
• Standardized testing and questionnaires provide comprehensive phenotypic characterization that allows for investigating the relation between behaviour and the neural basis of reading and language.
• Organized following the Brain Imaging Data Structure (BIDS) specification that enhances data usability for researchers by simplifying preprocessing pipelines and ensuring compatibility with widely used analysis tools.

## 2 Background

Functional magnetic resonance imaging (fMRI) data were gathered while children and adolescents completed visual word reading tasks with parametric manipulations, including localizer tasks that independently identified regions of interest associated with aspects of phonological and semantic processing. There have been published datasets on reading development in children that have investigated orthographic processing using spelling judgments, phonological processing using rhyming judgments, and semantic processing using meaning judgments in both the visual and auditory modalities [2]. There are also datasets that report data on the phonological processing of auditory, visual, and audio-visual words and pseudo-words using rhyming judgment tasks [3] and phonological processing in the visual modality using rhyming judgments along with arithmetic tasks [4]. The current dataset is like previous ones in that we use a visual word rhyming and a visual word meaning task with parametric manipulations. These similarities allow for replication analyses, but also mega-analyses combining different datasets. The parametric manipulations include words with conflicting spelling and sound information (e.g. pint-mint, grade-laid) versus non-conflicting information (e.g. lake-cake, press-list) in the rhyming tasks and words with high versus low word pair association (e.g. dog-cat, dish-plate) in the meaning tasks. These parametric manipulations are valuable in more directly measuring the implicating brain regions involved in certain processes.

The current dataset is unique in the use of localizer tasks to independently identify phonological and semantic processing without any oral or visual language input. The picture rhyming and picture meaning localizers only involve the presentation of two pictures to which the participants need to make a phonological or semantic judgment, respectively. Picture naming tasks have been used extensively in the literature to assess phonological and semantic processing in language [1,5]. The speech reading localizer involves the presentation of two videos of American English voiceless words (lip/mouth movements with no sound) and participants determine whether they match on specific dimensions (i.e., onset+vowel, rhyme). Speech reading has been extensively used in the literature to examine audio-visual processing in the brain [6] and its relation to reading and language [7]. The signed language localizer involves the presentation of two videos of ASL signs and participants must determine whether they match on certain dimensions (i.e., handshape, location, or movement). This task can be completed with no knowledge of signed language but will be particularly useful when examining its relation to reading in children who are deaf and hard of hearing (DHH) and use ASL. Studies have shown that signed language representations are important for reading development in DHH readers [8]. The triangle model posits that reading acquisition relies on a division of labor between orthographic, phonological, and semantic systems. This model has been investigated previously in typically developing children [9], but it is unknown how these mappings may change based on hearing variability. It could be that children who are DHH rely less on the mapping between orthography and phonology because of their altered auditory input, and thus, rely more on the mapping between orthography and semantics [10].

This dataset will be valuable to researchers examining the neuro-cognitive processes involved in reading and language. There have only been three pre-registered studies on this dataset, and none of them have been published yet in academic journals. One study examines whether the reliance on speech reading mechanisms in superior temporal cortex for visual word processing is related to reading skill [11]. The other investigates effective connectivity of the dorsal versus ventral inferior frontal cortex during visual word processing using dynamic causal modelling [12]. The final examines a gradient in the ventral occipito-temporal cortex for phonological versus semantic processing [13]. Given the limited amount of published work on this dataset, there are many opportunities for new discoveries. By sharing this dataset, we aim to provide a valuable resource for researchers studying the cognitive and neural basis of reading.

All dataset information in this article refers to dataset snapshot ds006239 (version 1.0.2) on OpenNeuro. Subsequent snapshots (e.g. version 1.0.3) add additional subjects and files; see the dataset CHANGES log for details.

## 3 Data Description

**Fig. 1:**
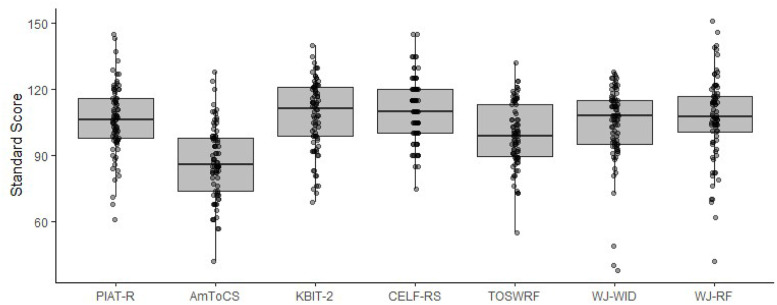
Distribution of scores for standardized measures–*Peabody Individual Achievement Test for Reading Comprehension (PIAT-R), American Test of Child Speech Reading (AmToCS), Kaufman Brief Intelligence Test Second Version (KBIT-2), Clinical Evaluations of Language Fundamentals (CELF-5)- Recalling Sentences, Test of Silent Word Reading Fluency (TOSWRF-2), and the Woodcock-Johnson III Tests of Achievement: Word Identification and Reading Fluency (WJ-WID and WJ-RF).* The box plots used in this figure represent the median values (dark black line at the center of each box). The lower and upper lines of each box correspond to the first and third quartiles (the 25th and 75th percentiles). The upper whisker extends from the box to the largest value no further than 1.5 times interquartile range (IQR, or distance between the first and third quartiles). The lower whisker extends from the box to the smallest value at most 1.5 times IQR. Exterior points represent outliers (1.5 times the interquartile range).

**Fig. 2:**
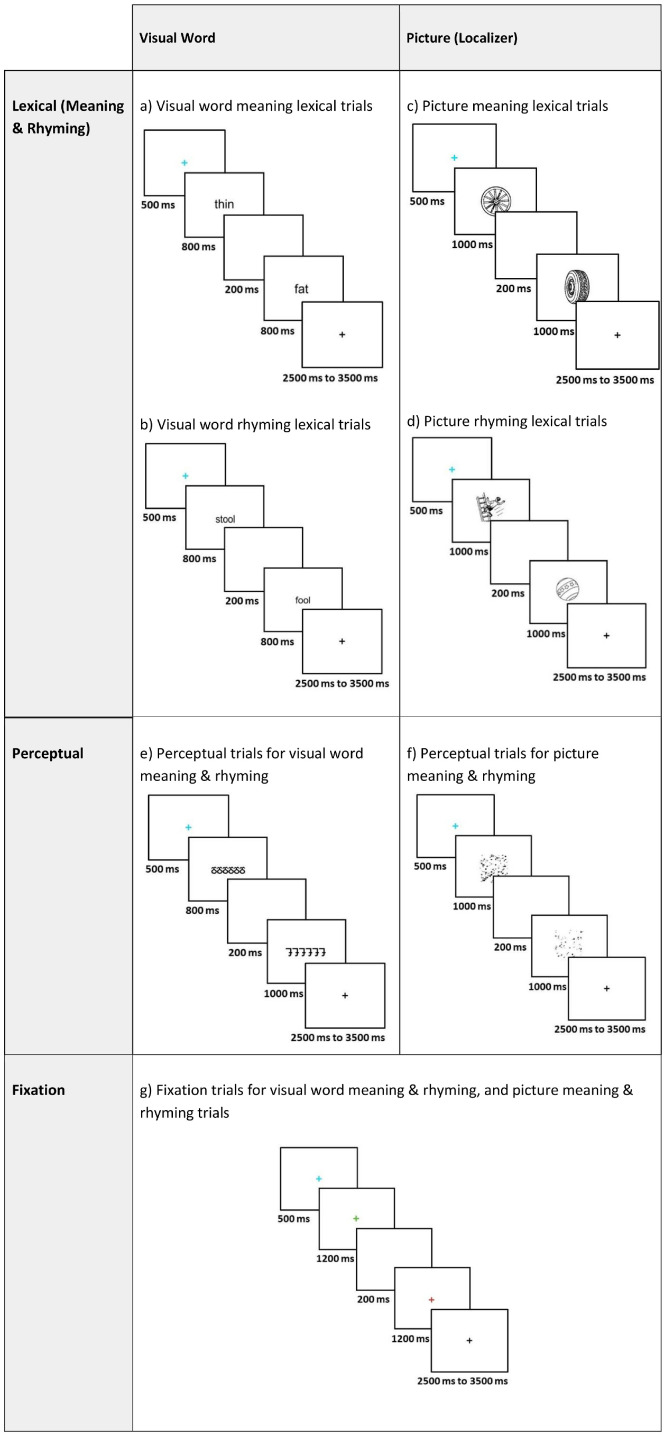
Trial type stimuli and timing. Illustration of the stimuli and timing for (a) visual word meaning task trials, (b) visual word rhyming task trials, (c) picture meaning task trials, (d) picture rhyming task trials (e) perceptual trials for visual word tasks, (f) perceptual trials for picture tasks, and (g) fixation trials for visual word and picture tasks.

**Fig. 3:**
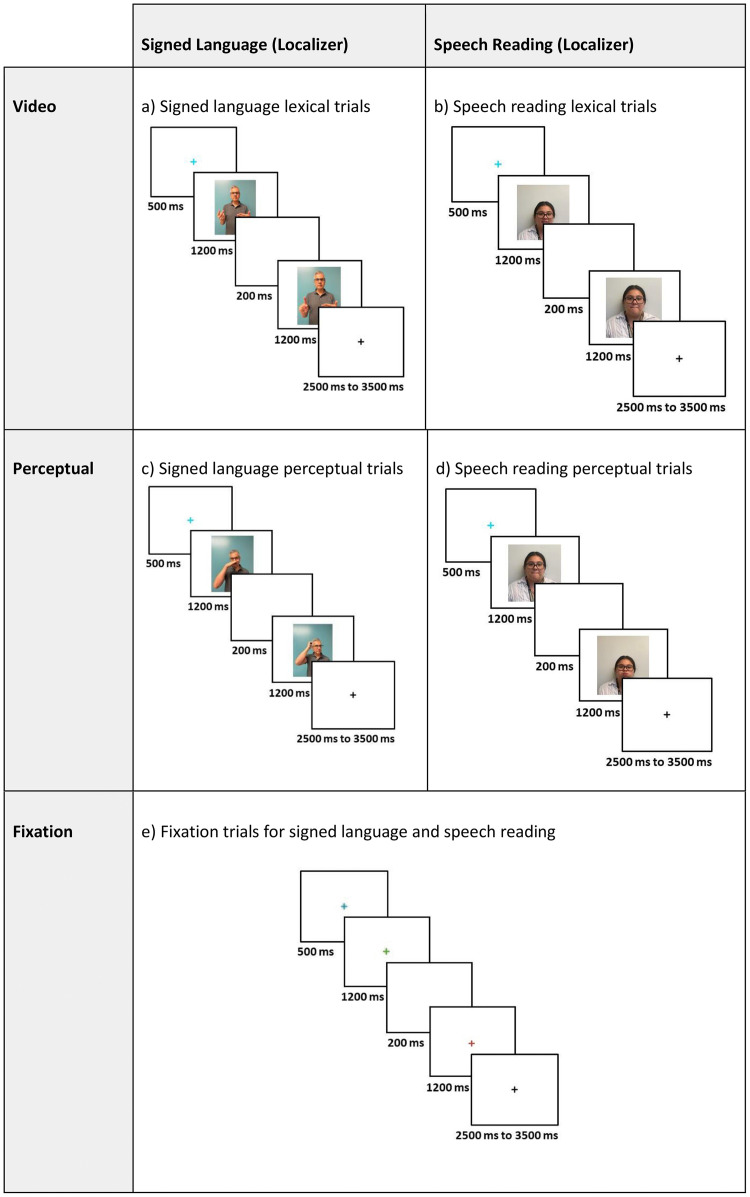
Trial type stimuli and timing. Illustration of the stimuli and timing for (a) signed language lexical trials (b) speech reading lexical trials, (c) perceptual trials for signed language, (d) perceptual trials for speech reading, and (e) fixation trials for both signed language and speech reading.

**Fig.4:**
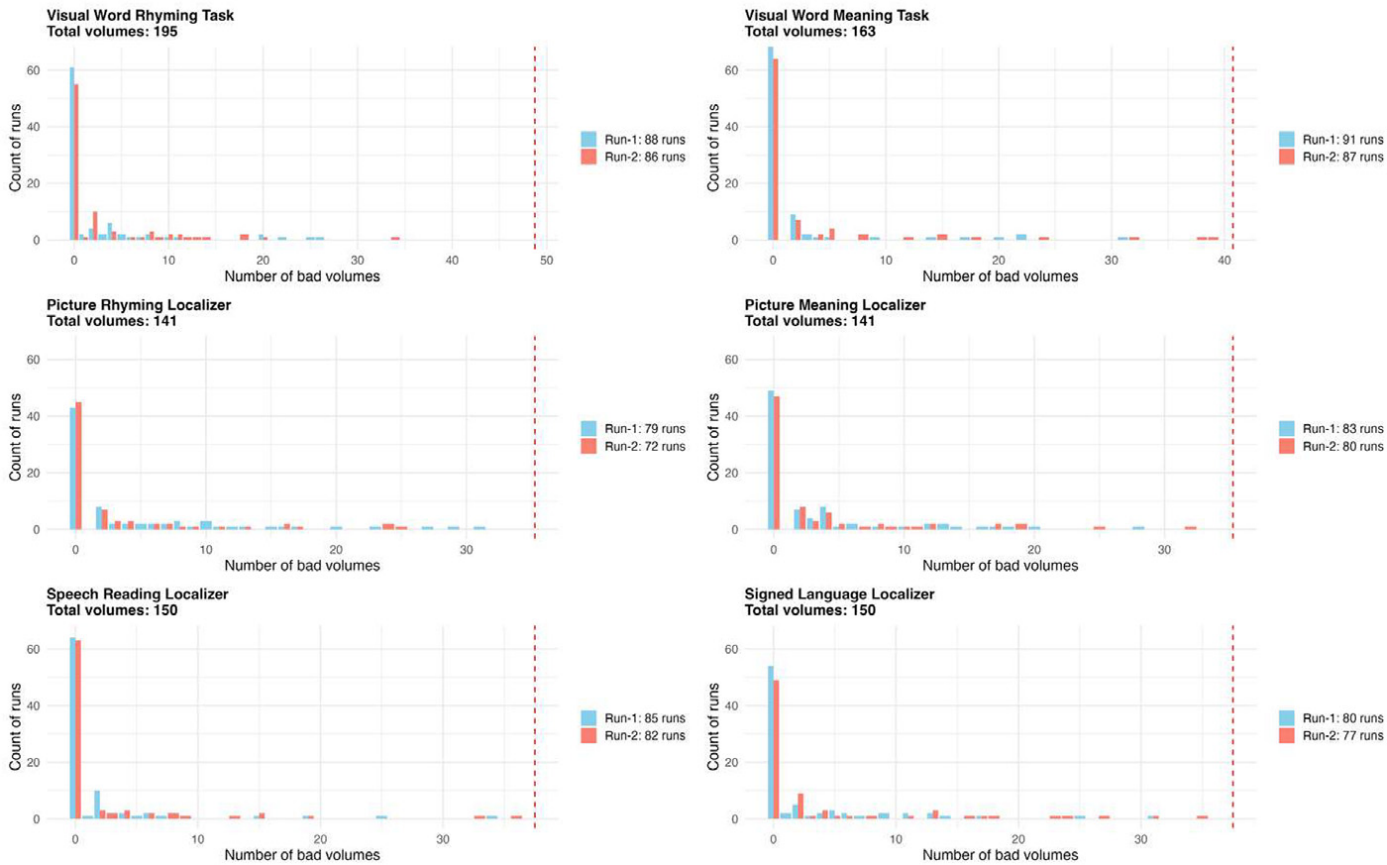
Histogram of motion artifact volumes across fMRI tasks and runs. Each bar indicates the number of runs with a given count of bad volumes, separated by run (Run-1 in blue, Run-2 in red). The red dashed vertical line denotes the exclusion threshold (25 % bad volumes); runs exceeding this threshold were excluded from the shared dataset. The number of included runs per task is shown on the right of each panel. Note that the number of runs may differ from the number of included participants due to missing runs, exclusion of runs with excessive movement, or the inclusion of duplicated good runs.

This dataset entitled “A fMRI neuroimaging dataset of word reading with semantic and phonological localizers in children and adolescents” is publicly available on OpenNeuro.org and is organized according to the Brain Imaging Data Structure specifications version 1.10.0 [14]. The dataset contains (1) raw T1-weighted structural images, (2) fMRI images acquired while participants completed six tasks including two reading and four localizer tasks, (3) diffusion tensor images, (4) behavioural data from fMRI tasks, (5) scores from standardized testing that measures hearing, reading, language, and cognitive abilities (6) participant language/reading background and demographics, and (7) all stimuli used for fMRI tasks. Included in this article, Table 1 describes the number of participants that completed each fMRI task, Table 2 summarizes participant questionnaire and survey information, Table 3 describes the standardized assessment measures collected, and Table 4 summarizes the accuracy and reaction time for fMRI tasks. Fig. 1 displays the distribution of scores for standardized measures, Fig. 2, Fig. 3 displays the fMRI task trial design, and Fig. 4 displays histograms of motion artifact volumes across fMRI tasks and runs.

**Table 1:** Number of participants who have completed one or more runs of each task is shown below. For details, the full subject-by-task matrix is available in the dataset version 1.0.3 or newer at /derivatives/task_counts_per_subject.xlsx on OpenNeuro.

fMRI Task	Total (*N* = 83)
Visual Word Rhyming Task	82
Visual Word Meaning Task	83
Picture Rhyming Localizer	77
Picture Meaning Localizer	81
Speech Reading Localizer	80
Signed Language Localizer	78

**Table 2:** Participant questionnaire and survey information.

Demographics			
Mean Age	Male (*N* = 41)	Female (*N* = 42)	Total (*N* = 83)
	12.4 (SD =1.9)	12.9(SD=1.9)	12.7(SD=2.0)
***N***			
**ADHD Rating**			
80th Percentile	4	8	12
90th Percentile	2	2	4
**Percentage of Participants**			
**Race**			
American Indian or Alaska Native	0 %	0 %	0 %
Asian	2.3 %	4.7 %	3.5 %
Black or African American	4.8 %	9.5 %	7.1 %
Native Hawaiian or Other Pacific Islander	0 %	0 %	0 %
White or Caucasian	83.3 %	66.7 %	75.0 %
Two or More Races	9.5 %	19.0 %	14.3 %
**Language**			
Monolingual	80.5 %	81.0 %	80.7 %
Multilingual	19.5 %	16.7 %	18.1 %
Choose not to answer	0 %	2.30 %	1.20 %
**Maternal Education**			
Less than High School	0 %	0 %	0 %
High School Graduate	2.4 %	7.1 %	4.8 %
Some College	0.0 %	16.7 %	8.4 %
2-year Degree	2.4 %	2.4 %	2.4 %
4-year Degree	48.8 %	23.8 %	36.1 %
Masters	29.3 %	35.7 %	33.7 %
Doctorate	9.8 %	9.5 %	9.6 %
Not provided or not applicable	7.3 %	4.8 %	4.8 %

**Table 3:** Tests and subtests completed across the complete session along with score type.

Measure	Test	Subtest	Scores
Hearing	Audiological Assessment	Otoscopy	Canal Status
		Pure-tone screening	Screening Results
		BKB-SIN	Signal-to-Noise Ratio for 50 % Correct (SNR-50)
Reading	Peabody Individual Achievement Test for Reading Comprehension (PIAT-R)		Raw Score
			Standard Score
	Test of Word Reading Fluency (TOSWRF-2)		Raw Score
			Standard Score
	Woodcock-Johnson III Tests of Achievement (WJ-III)	Word Identification	Raw Score
		Reading Fluency	Standard Score
	Derivational Morphology Task (DMORPH)		Accuracy
			Response Time
	Comprehensive Test of Phonological Processing (CTOPP-2)	Elision	Raw Score
		Blending Words	Scaled Score
		Phoneme Isolation	Composite Scores
	Clinical Evaluations of Language Fundamentals (CELF-5)	Recalling Sentences	Raw Score
		Sentence Comprehension	Standard Score
	American Test of Child Speech Reading (AmToCS)		Accuracy
			Response Time Standard Score
	Arizona-4 Tests of Articulation	Word Articulation	
			Raw Score
			Standard Score
ASL Phonological Awareness	American Sign Language Phonological Awareness Test (ASL-PAT)		Accuracy
			Average Repetitions
Problem-Solving	Kaufman Brief Intelligence Test (KBIT-2)		Raw Score
			Standard Score
Working Memory	Wechsler Intelligence Scale for Children (WISC-V)	Memory for Digits (backwards)	Span
			Total Score
	Corsi (backwards) Block-Tapping Test		Span
			Total Score

**Table 4:** Accuracy and response time summary for each experimental condition of each functional MRI task.

Task	Condition	Accuracy ( %, M, SD)	Response Time (s, M, SD)
Visual Word Rhyming Task	*O*+*P*+	89.1 (15.4)	1.626 (0.405)
	*O*+*P*-	74.8 (21.0)	1.811 (0.441)
	O-*P*+	85.4 (17.4)	1.688 (0.403)
	O-P-	90.7 (12.6)	1.677 (0.404)
Visual Word Meaning Task	HighY	90.6 (17.1)	1.594 (0.373)
	LowY	92.4 (13.8)	1.603 (0.36)
	UnrN	94.7 (10.8)	1.631 (0.388)
Picture Rhyming	PicY	70.7 (19.4)	1.982 (0.4)
	PicN	92.2 (16.5)	2.163 (0.428)
Picture Meaning	PicY	87.1 (13.7)	1.691 (0.307)
	PicN	92.0 (8.7)	1.742 (0.309)
Speech Reading	Onset	81.7 (20.9)	2.175 (0.393)
	Offset	69.1 (24.9)	2.121 (0.379)
	Diff	86.5 (13.6)	2.144 (0.353)
Signed Language	HS	77.5 (24.4)	2.169 (0.475)
	Loc	76.0 (22.7)	2.185 (0.433)
	Diff	86.4 (16.1)	2.159 (0.392)

*O+P+* = orthographically and phonologically similar; *O+P-* = orthographically similar and phonologically different; *O-P+* = orthographically different and phonologically similar; *O-P-* = orthographically nor phonologically different. *HighY* = high semantic association; *LowY* = low semantic association; *UnrN* = not semantically related. *PicY* = rhyming (in Picture Rhyming) or semantically related (in Picture Meaning) picture pairs; *PicN* = not rhyming (in Picture Rhyming task) or not semantically related (in Picture Meaning task) picture pairs.

*Onset* = shared onset phoneme; *Offset* = shared offset phoneme. *HS* = shared handshape; *Loc* = shared location; *Diff* = no phonological overlap (in Speech Reading and Signed Language).

Accuracy = percentage of correct responses; RT = response time in seconds. Values are reported as Mean (M) and Standard Deviations (SD). Subject-level accuracy and response time details for each task are included in the derivatives/acc_rt folder.

## 4 Experimental Design, Materials and Methods

### 4.1 Participants

This dataset includes 83 English-speaking participants ages 10–17 years (M*age* = 12.7, SD = 2.0, 42 female). Participants were recruited from the Nashville metro area and surrounding cities primarily through online advertisements (i.e. Facebook, Instagram, and Google Ads), community events, and fliers in schools and clinic offices.

Exclusionary criteria for this study included: (1) contraindications for MRI such as non-removal metal in or on the body (e.g. braces, permanent jewellery, implants, etc.), (2) claustrophobia, (3) preterm birth before 34 weeks, (4) birth complications requiring admission into a neonatal intensive care unit, (5) head injury requiring emergency medical evaluation, (6) uncorrected visual impairment, (7) hearing loss, (8) developmental or intellectual disabilities, or (9) medications affecting central nervous system processing. Parents completed an exclusionary survey as well as an exclusionary follow-up to determine eligibility. In the exclusionary follow-up, children completed the Mini Mental State Examination for Children (MMC) [15] and read a subset of the *Second Grade Dolch Words* [16]. Participants were required to score above 28 (out of 37) on the MMC and above 17 (out of 21) on the *Second Grade Dolch Words* to be eligible. A detailed description of the number of participants included in each fMRI experimental task is listed in Table 1.

### 4.2 Questionnaires and assessments

Parents of participants self-reported information about the child’s language access, educational background, handedness, attention/hyperactivity [17], parent socio-economic status (i.e. education and income), and reading practices in the home through a background questionnaire. Questions regarding language access involved parents identifying the perceived percentage of communicative signals in English and other languages that are received by the child at different time periods during development (i.e. birth to 36 months, most recent three years, and over their lifetime). Additionally, background questions included gathering information about language dominance and use in different environments (e.g. home vs. school) for children who identified as multilingual. This survey was completed during their first visit. Only a subset of the exclusionary survey and background survey are shared to maintain confidentiality. Table 2 summarizes some variables of the participant questionnaire and survey information including, race, language status (monolinguals versus multilinguals), and maternal education. We also calculated the number of participants who scored at or above the 80th and 90th percentile based on the ADHD Rating Scale-IV [17], thresholds that are often used for differentiating children with ADHD from those who do not have ADHD.

This dataset contains behavioural data from a variety of psycho-educational assessments, testing both linguistic and cognitive abilities. These assessments include the Peabody Individual Achievement Test for Reading Comprehension (PIAT-R) [18], Derivational Morphology Task (DMORPH) [19], Woodcock-Johnson III Tests of Achievement (WJ-III) Word Identification (WJ-WID) and Reading Fluency (WJ-RF) [20], Test of Word Reading Fluency (TOSWRF-2) [21], Clinical Evaluations of Language Fundamentals (CELF-5) [22], American Test of Child Speech Reading (AmToCS) [23], Comprehensive Test of Phonological Processing (CTOPP-2) [24], Arizona test of Articulation Version 4 (Arizona-4) [25], American Sign Language Phonological Awareness Test (ASL-PAT) [26] the Kaufman Brief Intelligence Test Second Version (KBIT-2) [27], Wechsler Intelligence Scale for Children (WISC-V) memory for digit span- backwards [28], and Corsi (backwards) block-tapping test [29]. Table 3 provides an overview of the tests and score types and Fig. 1 depicts the distributions for the standardized measures. Standard scores are not provided for the WISC-V memory for digit span test since only the backwards portion was administered. All assessments were administered starting with the PIAT-R [18]. The PIAT-R [18] was administered either in the lab or over Zoom, and all other tests were completed in-person. Participants also completed a hearing test consisting of an otoscopy check, pure-tone screening at 20 dB, and the BKB-speech in noise test (BKB-SIN) [30]. Session duration lasted from three hours to six hours and additional tests were administered in a separate session if needed. All behavioural assessments for each participant were completed within a timeframe of several days to a few months, depending on scheduling availability.

### 4.3 Practice imaging

Participants completed an hour-long practice session prior to each of their imaging sessions. During this session, participants first were familiarized with tasks through a PowerPoint presentation and given a few example trials. Then, they completed full-length practice versions of the tasks inside a Psychology Software Tools mock scanner. Stimuli for practice sessions differed from the stimuli in the in-scanner tasks. This practice served to simulate scanner conditions (e.g., scanner noises, practice with button box, etc.) for enhanced understanding of the tasks, procedures, and to reduce anxiety surrounding the scanner environment.

### 4.4 Image acquisition

**Structural MRI.** T1-weighted Turbo Field Echo (TFE) images were acquired with the following parameters: TR = 8.9 ms, TE = 4.6 ms, field of view (FOV) = 256 × 256 mm, matrix size = 256 × 256, slice thickness = 1 mm, voxel size = 1 mm isotropic, flip angle = 8°, acceleration factor (SENSE) = 2, acquisition mode = Cartesian, and bandwidth = 217 Hz/px. Imaging was performed with a 32-channel head coil in the supine position.

**Functional MRI.** Blood oxygen level-dependent (BOLD) signals were acquired using T2-weighted single-shot echo-planar imaging (EPI)* with the following parameters: TR = 2000 ms, TE = 30 ms, field of view = 216 mm, matrix size = 96 × 94, slice thickness = 2.25 mm with no gap, number of slices = 54, voxel size = 2.25 mm isotropic, flip angle = 72°, and multi-band acceleration factor = 2. Slices were acquired in an interleaved order from foot to head.

**Diffusion Tensor Imaging**. Diffusion tensor imaging were collected using single-shot echo planar imaging (EPI) with the following parameters: TR ranges from 3297 ms to 5842 ms, TE = ranges from 51 ms to 66 ms, field of view = 220 mm, matrix size = 112 × 110, slice thickness = 2 mm, number of slices = 72, voxel size = 1.96 × 1.96 × 2 mm, multi-band acceleration factor = 2, b-value1 = 0 s/mm², b-value2 = 1000 s/mm², diffusion directions = 64. Slices were acquired in ascending order from foot to head. Subject level parameters summary is included in the derivatives folder.

**TOPUP Field Mapping.** TOPUP Field Mapping were collected using spin-echo-planar images (EPI) with the following parameters: TR = 1719 ms, TE = 34 ms, field of view (FOV) = 216 × 216 mm, matrix size = 96 × 94, bandwidth = 2150 Hz/px, slice thickness = 2.25 mm with no gap, number of slices = 54, voxel size = 2.25 mm isotropic, flip angle = 90°, and EPI factor = 47. Parallel imaging was applied with an acceleration factor (SENSE) = 2. Images were collected with reversed phase-encoding directions—anterior-to-posterior (APP) and posterior-to-anterior (APA)—for subsequent susceptibility distortion correction using FSL TOPUP. Subject level parameters summary is included in the derivatives folder.

### 4.5 Functional MRI tasks

**Visual Word Rhyming Task**. The reading rhyming task was designed to visually present participants with two monosyllabic English words and asked to make a rhyming judgement. Word pairs were categorized into four experimental conditions: orthographically similar and phonologically similar (*O* + *P*+), orthographically different and phonologically similar (O-*P*+), orthographically similar and phonologically different (*O* + *P*-), and orthographically different and phonologically different (O-P-). Participants were instructed to respond ‘yes’ using their right index finger for rhyming conditions (*O* + *P*+ and O-*P*+) and ‘no’ using their right middle finger for the non-rhyming conditions (*O* + *P*- and O-P-). In addition to the four experimental conditions, the task included a perceptual control condition (Perc), in which participants responded ‘yes’ when presented with a pair of matched false font symbols and ‘no’ when the symbols were not matched. The task also included a fixation baseline condition (Fix), where participants responded ‘yes’ when they saw a pair of crosses with the same color and ‘no’ when the crosses were different colors. The task consisted of 144 trials, divided into two separate 72-trial runs with 12 trials per condition per run. The order of the trials was pseudorandomized, ensuring that no more than three trials of the same conditions appeared consecutively. In each trial, the first stimulus was presented for 800 ms, followed by a 200 ms blank inter-stimulus-interval, and a second 800 ms for the text stimulus. The response window showed a black fixation cross and varied from 2500 to 3500 ms, with one-third-of trials having each of the following inter-trial intervals: 2500 ms, 3000 ms, and 3500 ms. An additional blue fixation cross was shown for 500 ms, as an indication that the next trial would soon begin. Each run of the reading rhyming task lasted 6 min and 44 s.

Words were carefully chosen to dissociate orthographic (O; shared letters) and phonological (P; shared sounds) overlap. The word stimuli used in the current study were adapted from a corpus of 2998 monosyllabic words [31]. Overlap percentage of letters between the pairs of words were computed for each of the four experimental conditions. Overlap percentage of letters was defined as the number of letters overlapped between the prime and the target divided by the average number of letters between the prime and the target. The averages were: *O* + *P*-: 71.3 %; O-*P*+: 46.1 %; *O* + *P*+: 68.8 %; O-P-: 34.6 %. Overlap percentage of phonemes between the prime and the target were computed for each of the four conditions. Overlap percentage of phonemes was defined as the number of phonemes overlapped between the prime and the target divided by the average number of phonemes between the prime and the target. The averages were: *O* + *P*-: 30.6 %; O-*P*+: 62.1 %; *O* + *P*+: 63.2 %; O-P-: 30.3 %.

All stimulus words were monosyllabic, concrete English nouns with only one morpheme, selected to systematically vary in orthographic and phonological overlap. Each word appeared only once across the experiment, serving as either a prime or a target. Primes and targets were matched across key lexical variables, including word length (*M* = 4.61 letters, SD = 0.79), log-transformed HAL frequency (*M* = 8.92, SD = 1.39), lexical decision naming accuracy (*M* = 0.97, SD = 0.03), and bigram frequency (*M* = 10,467, SD = 4836) from the English Lexicon Project [32]. Child-written word frequency (*M* = 9.07, SD = 16.07) was calculated based on corpus norms described in [31]. These variables were comparable across the four experimental conditions, with no systematic differences observed in word length, naming accuracy, bigram frequency, or child-written word frequency (ps > 0.23). A small difference in lexical frequency was present across conditions (F(3188) = 2.68, *p* = .048), though this was not a feature of the task design and likely reflects natural limitations in word availability when jointly manipulating phonological and orthographic similarity. Sublexical properties were evaluated using phonological and orthographic consistency metrics [31,33]. Phonological consistency was defined as the proportion of phonological friends—words that share both rime spelling and pronunciation (e.g., cat, bat)—relative to the total number of phonological friends and phonological enemies. Phonological enemies are words that share the same rime spelling but differ in pronunciation (e.g., lead [liːd] vs. dead [dɛd]). Similarly, orthographic consistency was defined as the proportion of orthographic friends—words that share both the pronunciation and spelling of the rime (e.g., fight, night)—relative to the total number of orthographic friends and orthographic enemies. Orthographic enemies are words with the same pronunciation but different spellings (e.g., see, sea). Across all items, mean phonological consistency was 0.71 (SD = 0.32) and orthographic consistency was 0.53 (SD = 0.27). As expected based on the condition structure, these sublexical features varied significantly across conditions (phonological consistency: F(3188) = 20.69, *p* < .001; orthographic consistency: F(3188) = 4.35, *p* = .005), as did the number of phonological (*M* = 3.05, SD = 4.18; F(3188) = 11.63, *p* < .001) and orthographic enemies (*M* = 6.32, SD = 4.98; F(3188) = 6.25, *p* < .001). These differences reflect the intended manipulation of rime-level similarity and dissimilarity across experimental conditions.

**Visual Word Meaning Task.** The reading meaning task was designed to visually present participants with two monosyllabic English words and asked them to decide if they relate to each other. Word pairs were categorized into three experimental conditions: high semantic association (HighY), low semantic association (LowY), and not semantically related (UnrN). Participants were instructed to respond ‘yes’ for semantically related conditions (HighY and LowY) and ‘no’ for the not semantically related condition (UnrN). In addition to the three experimental conditions, the task included a perceptual control condition (Perc), in which participants responded “yes” when presented with a pair of matched false font symbols and ‘no’ when the symbols were not matched. The task also included a fixation baseline condition (Fix), where participants responded “yes” when they saw a pair of crosses with the same color and ‘no’ when the crosses were different colors. The task consisted of 120 trials, divided into two separate 60-trial runs with 12 trials per condition per run. The order of the trials was pseudorandomized, ensuring that no more than three trials of the same conditions appeared consecutively. In each trial, the first stimulus was presented for 800 ms, followed by a 200 ms blank inter-stimulus-interval, and a second 800 ms for the text stimulus. The response window showed a black fixation cross and varied from 2500 to 3500 ms, with one-third-of trials having each of the following inter-trial intervals: 2500 ms, 3000 ms, and 3500 ms. An additional blue fixation cross was shown for 500 ms, as an indication that the next trial would soon begin. Each run of the reading meaning task lasted 5 min and 40 s.

All stimulus words were monosyllabic, concrete English nouns with only one morpheme. Each word appeared only once across the experiment, serving as either a prime or a target. Primes and targets were matched across key lexical variables, including word length (*M* = 5.02 letters, SD = 1.19), log-transformed HAL frequency (*M* = 9.04, SD = 1.64), and bigram frequency sum (*M* = 14,749, SD = 8961), all derived from the English Lexicon Project [32]. Child-written word frequency (*M* = 18.84, SD = 42.15) was estimated using corpus norms described in [31]. These lexical variables were comparable across the three experimental conditions, with no significant differences observed in word length, frequency, bigram frequency, or child-written frequency (ps > 0.34). Semantic association strength was assessed using forward association norms from the University of South Florida Free Association Norms [34], which reflect the proportion of participants who produced the target word in response to the prime in free association tasks. As intended by design, association strength was significantly higher in the HighY condition (*M* = 0.60, SD = 0.11) than in the LowY condition (*M* = 0.30, SD = 0.11), Welch’s t (45.96) = 9.14, *p* < .001. Unrelated (UnrN) pairs were selected such that they had no available forward association in the norming database. These results confirm the successful manipulation of semantic relatedness across conditions.

**Picture Rhyming.** The picture rhyming localizer was designed to visually present participants with two pictures corresponding to English words. Participants were asked to mentally label the objects in English and make a rhyming judgement. Picture pairs were categorized into two experimental conditions: rhyming (PicY), and not rhyming (PicN). Participants were instructed to respond ‘yes’ for rhyming condition (PicY) and ‘no’ for the not rhyming condition (PicN). In addition to the two experimental conditions, the task included a perceptual control condition (Perc), in which participants responded “yes” when presented with a pair of matched visually scrambled pictures and ‘no’ when the pictures were not matched. The task also included a fixation baseline condition (Fix), where participants responded “yes” when they saw a pair of crosses with the same color and ‘no’ when the crosses were different colors. The task consisted of 96 trials, divided into two separate 48-trial runs with 12 trials per condition per run. The order of the trials was pseudorandomized, ensuring that no more than three trials of the same conditions appeared consecutively. In each trial, the first stimulus was presented for 1000 ms, followed by a 200 ms blank inter-stimulus-interval, and a second 1000 ms stimulus. The response window showed a black fixation cross and varied from 2500 to 3500 ms, with one-third-of trials having each of the following inter-trial intervals: 2500 ms, 3000 ms, and 3500 ms. An additional blue fixation cross was shown for 500 ms, as an indication that the next trial would soon begin. Each run of the picture rhyming localizer lasted 4 min and 56 s.

Stimuli were selected from the International Picture Naming Project (IPNP) database [35], which was filtered for monosyllabic words that began with a consonant, and further filtered for high percent naming agreement (i.e., IPNP’s elex1 > 0.80). No words are repeated within or across picture tasks (Picture Meaning Localizer and Picture Rhyming Localizer). All items were matched across rhyming and not rhyming conditions on key lexical characteristics, including word length (*M* = 4.25, SD = 0.79), log-transformed HAL frequency (*M* = 9.29, SD = 1.43) derived from the English Lexicon Project [32], bigram frequency (*M* = 11,064, SD = 6401), and IPNP’s elex1 (*M* = 0.94, SD = 0.06). Phonological frequency was computed as the average positional probability of adjacent phoneme pairs (i.e., biphones), using values obtained from the University of Kansas Phonotactic Probability Calculator [36]. No significant differences were observed across the rhyming and non-rhyming conditions on any of these measures (ps > 0.07) [32].

**Picture Meaning.** The picture meaning localizer was designed to visually present participants with two images and asked them to judge whether the two images are semantically related. Picture pairs were categorized into two experimental conditions: semantically related (PicY), and not semantically related (PicN). Participants were instructed to respond ‘yes’ for semantically related condition (PicY) and ‘no’ for the not semantically related condition (PicN). In addition to the two experimental conditions, the task included a perceptual control condition (Perc), in which participants responded “yes” when presented with a pair of matched visually scrambled pictures and ‘no’ when the pictures were not matched. The task also included a fixation baseline condition (Fix), where participants responded “yes” when they saw a pair of crosses with the same color and ‘no’ when the crosses were different colors. The task consisted of 96 trials, divided into two separate 48-trial runs with 12 trials per condition per run. The order of the trials was pseudorandomized, ensuring that no more than three trials of the same conditions appeared consecutively. In each trial, the first stimulus was presented for 1000 ms, followed by a 200 ms blank inter-stimulus-interval, and a second 1000 ms stimulus. The response window showed a black fixation cross and varied from 2500 to 3500 ms, with one-third-of trials having each of the following inter-trial intervals: 2500 ms, 3000 ms, and 3500 ms. An additional blue fixation cross was shown for 500 ms, as an indication that the next trial would soon begin. Each run of the picture meaning localizer lasted 4 min and 56 s.

Stimuli were selected from the International Picture Naming Project (IPNP) database [35], which was filtered for monosyllabic words that began with a consonant, and further filtered for high percent naming agreement (i.e., IPNP’s elex1 > 0.80). No words are repeated within or across picture tasks (Picture Meaning Localizer and Picture Rhyming Localizer). All items were matched across conditions on key lexical characteristics, including word length (*M* = 4.21, SD = 0.79), bigram frequency (*M* = 9911, SD = 6213), and IPNP’s elex1 (*M* = 0.94, SD = 0.07), with no significant differences observed (ps > 0.07). However, a significant difference in log-transformed HAL frequency (*M* = 9.31, SD = 1.41) was present between conditions (F (1,94) = 7.07, *p* = .009). This difference likely reflects constraints in stimulus availability given the requirement to manipulate semantic relatedness while balancing visual features. Lexical characteristics were drawn from the English Lexicon Project [32].

**Speech Reading.** The speech reading localizer was designed to visually present participants with two sequential silent face videos of a man or woman saying (without sound) a single monosyllabic American English word, and asked them to judge if the two words in the videos are similar. Video pairs were categorized into three experimental conditions: phonological onset (Onset), phonological offset (Offset), and no phonological overlap (Diff). Participants were instructed to respond ‘yes’ for phonologically similar conditions (Onset and Offset) and ‘no’ for the not phonologically similar condition (Diff). In addition to the three experimental conditions, the task included a perceptual control condition (Perc), in which participants responded “yes” when presented with a pair of matched nonsense facial gestures (e.g., tongue out) and ‘no’ when the facial gestures were not matched. The task also included a fixation baseline condition (Fix), where participants responded “yes” when they saw a pair of crosses with the same color and ‘no’ when the crosses were different colors. The task consisted of 96 trials, divided into two separate 48-trials runs. Each run contained 6 trials from the Onset and Offset conditions, and 12 trials each from the Diff, Perc, and Fix conditions. The order of the trials was pseudorandomized, ensuring that no more than three trials of the same conditions appeared consecutively. In each trial, the first stimulus was presented for 1200 ms, followed by a 200 ms blank inter-stimulus-interval, and a second 1200 ms stimulus. The response window showed a black fixation cross and varied from 2500 to 3500 ms, with one-third-of trials having each of the following inter-trial intervals: 2500 ms, 3000 ms, and 3500 ms. An additional blue fixation cross was shown 500 ms, as an indication that the next trial would soon begin. Each run of the speech reading localizer lasted 5 min and 14 s.

Each video depicted a talker articulating a monosyllabic American English word, selected to systematically vary in phonological overlap while controlling for lexical properties. All word stimuli were CVC-structured and visually matched based on shared visemes, with similar items sharing either the initial consonant-vowel (CV) or final vowel-consonant (VC) segment, and dissimilar items sharing no visemes. All items had a fixed word length of 3 letters and were matched across conditions on log-transformed HAL frequency (*M* = 9.62, SD = 1.74) and bigram frequency (*M* = 2741, SD = 1874). All words had above-average written log frequency. No significant differences were observed across the Onset, Offset, and Diff conditions (ps > 0.08). Lexical characteristics were drawn from the English Lexicon Project [32].

**Signed Language.** The ASL phonology localizer was designed to visually present participants with two videos of either a male or female signer producing common ASL signs and asked them to judge if they are similar or different. Participants are instructed similar signs can be ASL signs with two of three shared phonological parameters: handshape, location and/or movement. Movement was controlled and only dissimilar handshape and location were included in the experimental trials. Video pairs were categorized into three conditions: shared handshape (HS), shared location (Loc), and no phonological overlap (Diff). Participants were instructed to respond ‘yes’ for phonologically similar conditions (HS and Loc) and ‘no’ for the non-phonologically similar condition (Diff). In addition to the two experimental conditions, the task included a perceptual control condition (Perc), in which participants responded “yes” when presented with a pair of matched grooming gestures (e.g., touch hair) and ‘no’ when the gestures are not matched. The task also included a fixation baseline condition (Fix), where participants responded “yes” when they saw a pair of crosses with the same color and ‘no’ when the crosses are different colors. The task consisted of 96 trials, divided into two separate 48-trials runs. Each run contained 6 trials from the HS and Loc conditions, and 12 trials each from the Diff, Perc, and Fix conditions. The order of the trials was pseudorandomized, ensuring that no more than three trials of the same conditions appeared consecutively. In each trial, the first stimulus was presented for 1200 ms, followed by a 200 ms blank inter-stimulus-interval, and a second 1200 ms video stimulus. The response window showed a black fixation cross and varied from 2500 to 3500 ms, with one-third-of trials having each of the following inter-trial intervals: 2500 ms, 3000 ms, and 3500 ms. An additional blue fixation cross was shown for 500 ms, as an indication that the next trial would soon begin. Each run of the ASL phonology localizer lasted 5 min and 14 s.

Stimuli were filtered to vary in phonological similarity and were matched across conditions on several lexical and phonological properties. None of the items were initialized signs, or fingerspelling loan signs, and all signs had English translation equivalents and did not include facial expressions or mouthing. No signs were repeated within the ASL Phonology Localizer task. Items were matched across the handshape (HS), location (Loc), and unrelated (Diff) conditions on log-transformed HAL frequency (*M* = 10.25, SD = 1.61) derived from English Lexicon Project [32], ASL subjective frequency (*M* = 4.77, SD = 1.01), and ASL iconicity (*M* = 2.59, SD = 1.34). ASL subjective frequency reflects native and early-exposed signers’ rated familiarity with each sign, and ASL iconicity refers to how strongly a sign’s form resembles its meaning, based on combined ratings from deaf and hearing individuals. Both measures were drawn from the ASL-LEX database [37]. No significant differences were observed across the phonological conditions on any of these measures (p’s > 0.47) [32].

### 4.6 De-identification and quality control

Participant information was anonymized by removing identifying information from the free response questions in the surveys as well as shifting birthdates and testing dates for the standardized assessments and in-scanner sessions. The birthdate was shifted by adding a random number of days ranging from 1 to 365 to the original date and then subtracting 100 years. The time between the birthdate and the sessions was consistent within participants. Shifted acquisition dates are available in the “partcipants.tsv” file at the root level of the dataset.

All standardized assessments were coded twice by trained research team members who then compared items and made joint decisions on final scores. If there was any disagreement that could not be resolved, a third team member was recruited to make the final judgement. Upon curation of the dataset, all scores were reviewed to ensure no data entry errors had occurred.

Neuroimaging data was converted from standard DICOM to NIFTI format using dcm2niix version 1.0.20230411. During conversion dcm2niix extracted necessary scanning parameters from the dicom header and saved this information in an accompanying sidecar json file to each nifti image. Facial features were scrubbed from all T1-weighted images using pydeface (https://github.com/poldracklab/pydeface) in order to de-identify the anatomical images. All T2-weighted images were evaluated for in-scanner movement using the ArtRepair toolbox [38]. Within each run, volumes that had a greater than 1.5 mm volume-to-volume motion, or global signal intensity deviation greater than 4 % were marked as bad volumes. For each participant the functional fMRI task runs containing greater than 25 % of bad volumes were removed from the shared dataset (see histograms of all the included good runs with <25 % bad volumes in Fig. 4). After facial feature removal and the exclusion of runs with excessive motion, all T1- and T2-weighted images were reviewed with the MRI Quality Control tool (MRIQC), version 0.15.2 [39]. MRIQC reports are included in the derivatives/mriqc folder.

## Limitations

There are some notable limitations of this dataset. First, this dataset is biased towards proficient readers. Based on norms for our reading comprehension screener, the PIAT-R, (out of 83 participants) 56 participants performed above the population mean and 32 performed above one standard deviation above of the mean. This bias may be important when considering the generalizability of potential findings. Second, there are differences in the number of scans per task, namely, the picture rhyming localizer only has 77 participants with successful scans. The contrast in the number of useable scans by task is mainly due to known difficulties in scanning pediatric populations such as incompatibility with the scanner environment or fatigue, as the tasks that have a reduced number of scans were administered toward the end of last day of acquisition. Lastly, in our visual word rhyming task and picture meaning localizer there is a significant difference in lexical frequency across experimental conditions (*p*<.05). Thus, possible observed behavioural signatures of increased processing difficulty during in-scanner tasks may not only be due to incongruence between linguistically-manipulated components, but also may be partially attributed to word frequency effects.

## Ethics Statement

This research was approved by the Vanderbilt Institutional Review Board (IRB #171,739) and all subjects provided informed consent.

## Credit Author Statement

**Jiuru Wang:** Writing - Original Draft, Writing - Review & Editing, Data Curation, Data Sharing, Software, Validation, Visualization, Quality Control, Resources; **Avery Vess:** Writing - Original Draft, Writing - Review & Editing, Investigation, Software, Validation, Visualization, Project administration, Data Curation; **Avantika Mathur:** Writing - Review & Editing, Software, Resources, Data Curation, Supervision; **Neelima Wagley:** Writing - Review & Editing, Software, Methodology, Validation, Data Curation, Investigation, Project administration, Investigation; **David Quinto-Pozos:** Writing - Review & Editing, Resources, Methodology; **James Booth:** Writing - Review & Editing, Conceptualization, Methodology, Supervision, Funding acquisition.

## Data Availability

OpenNeuroA fMRI neuroimaging dataset of word reading with semantic and phonological localizers in children and adolescents (Original data).
